# Assessing changes in costs of maternal postpartum services between 2013 and 2014 in Burkina Faso

**DOI:** 10.1186/s12939-019-1064-5

**Published:** 2019-10-15

**Authors:** Danielle Yugbaré Belemsaga, Anne Goujon, Olivier Degomme, Tchichihouenichidah Nassa, Els Duysburgh, Seni Kouanda, Marleen Temmerman

**Affiliations:** 1 Biomedical and Public Health Department, Institut de Recherche en Sciences de la Santé, 03 B. P 7192, Ouagadougou, 03 Burkina Faso; 2 Wittgenstein Centre for Demography and Global Human Capital (IIASA, VID/OAW, WU), Vienna, Austria; 3 International Centre for Reproductive Health, Faculty of Medicine and Health Sciences, Department of public health and primary care, Ghent University, Ghent, Belgium; 4 Direction générale des études et des statistiques sectorielles (DGESS), Ministère de la santé, Ouagadougou, Burkina Faso; 5 African Institute of Public Health, Ouagadougou, Burkina Faso; 6 Centre of Excellence in Women and Child Health, Aga Khan University, Nairobi, Kenya

**Keywords:** Postpartum care, Maternal and infant health, Health service costs, Household costs, Integration of services, Burkina Faso

## Abstract

**Introduction:**

In Africa, a majority of women bring their infant to health services for immunization, but few are checked in the postpartum (PP) period. The *Missed opportunities for maternal and infant health* (MOMI) EU-funded project has implemented a package of interventions at community and facility levels to uptake maternal and infant postpartum care (PPC). One of these interventions is the integration of maternal PPC in child clinics and infant immunization services, which proved to be successful for improving maternal and infant PPC.

**Aim:**

Taking stock of the progress achieved in terms of PPC with the implementation of the interventions, this paper assesses the economic cost of maternal PPC services, for health services and households, before and after the project start in Kaya health district (Burkina Faso).

**Methods:**

PPC costs to health services are estimated using secondary data on personnel and infrastructure and primary data on time allocation. Data from two household surveys collected before and after one year intervention among mothers within one year PP are used to estimate the household cost of maternal PPC visits. We also compare PPC costs for households and health services with or without integration. We focus on the costs of the PPC intervention at days 6–10 that was most successful.

**Results:**

The average unit cost of health services for days 6–10 maternal PPC decreased from 4.6 USD before the intervention in 2013 (Jan-June) to 3.5 USD after the intervention implementation in 2014. Maternal PPC utilization increased with the implementation of the interventions but so did days 6–10 household mean costs. Similarly, the household costs increased with the integration of maternal PPC to BCG immunization.

**Conclusion:**

In the context of growing **r**eproductive health expenditures from many funding sources in Burkina Faso, the uptake of maternal PPC led to a cost reduction, as shown for days 6–10, at health services level. Further research should determine whether the increase in costs for households would be deterrent to the use of integrated maternal and infant PPC.

## Introduction

Burkina Faso is a West African low income country with a gross domestic product (GDP) per capita of 650 United States Dollar (USD) in 2016 [1]. Health expenditures accounted for 5.6% of GDP in 2013 and 6.2% in 2015. Household out–of-pocket spending as percent of total health expenditures increased from 28.2% in 2013 to 34.2% in 2015 [2]. Since 2000, free health care has been extended to more and more interventions for the population of Burkina Faso [3]. In the sector of Reproductive Maternal Newborn and Child Health (RMNCH) the exemption policies have been affecting for instance [2] 1) antenatal and under-five-year care since 2002; 2) vaccinations under the Expanded Program on Immunization (EPI) for infants up to the age of 11 months and for pregnant women since 2006; 3) services provided under the national Emergency Obstetric and Newborn Care (EmONC) program and childbirth delivery subsidies from 2006 to 2016; 4) a decree providing free health care for children under the age of five and women since early 2016 [2]; 5) subsidies for family planning (FP) since 2009. Because of these policies, most services are free e.g., antenatal care (ANC), EPI, EmONC and others e.g. FP are subsidized. Moreover, in 2015, the government adopted a law to ensure universal health coverage for all Burkinabe, although it has not yet been enacted; and created in March 2018 the National Fund for Universal Health Insurance (CNAMU). Several other large initiatives have been introduced since 2015 such as a Performance Based Financing pilot program with the aim notably of upgrading the provision of maternal health services [4–6].

The postpartum (PP) period includes the first six weeks (equivalent to approximately 42 days) following childbirth [7]; the late PP period comprises the period between 42 days and a year after delivery [8, 9]. Postpartum care (PPC) encompasses the management of the mothers, infant and newborn during the PP period [7].

The MOMI project over the period 2011–2015 aimed at reducing maternal and newborn mortality and morbidity within the PP period by upgrading PPC through a combined package of facility and community interventions in four African countries (Burkina Faso, Kenya, Malawi and Mozambique) [8]. The three-country specific interventions implemented from September 2013 to December 2015 in the Kaya health district in Burkina Faso were the following: (i) Female community health workers (CHWs) who were traditionally accompanying women for delivery in the health facilities (HFs) would be additionally supporting mothers and infants during the PP period; (ii) Providing immediate PPC in HFs was enhanced with a focus on the detection and management of PP haemorrhage and sepsis; (iii) PPC (including FP counselling and provision) for the mother and infant was integrated to child vaccination in HFs [8]*.* Previous studies have evaluated the potential impact of the interventions and found an improved uptake of PPC in quantity and quality [10]. In Kaya Health district, the improvements were particularly visible at days 6–10 PPC [11]*.*

There is however lack of evidence on the costing effect of the uptake of maternal PPC both at health services and households levels while part of the rationale for the integration was its cost-effectiveness for both units of analysis. In some other context, Nolte found that interventions such as integrated care could contribute to improve health outcomes and to reduce health service costs notably in hospitals [13]. On one hand, raising quality would lead to an increase in the use of health services that would presumably inflate the overall costs of service delivery (more supplies, more time spent for each visits, etc.). However, at the household and community level, the cost in monetary and time terms may decline as a result of the rationalization of care delivery. In addition, the transport and the indirect cost should be reduced due to the integration, as mothers should come once for both infant immunization and maternal PP checks-ups.

It is worth noting that PPC was not free of charge for households during the intervention implementation. The gratuity of PPC started formally in 2016 with the introduction of the policy on free health care for children under the age of five and women.

Our paper aimed at assessing the economic costs of maternal PP services, both for health services and households before and after the implementation of the interventions seeking to uptake maternal PPC services in Kaya health district (Burkina Faso). Since the cost of the PP visit at the first 48 h, at days 6–10 or onwards during weeks 6–8 should be circa the same as they are comparable in terms of duration and medical supplies used, we focus on the intervention at days 6–10, that was the most successful. We compare the costs before the intervention and after one-year intervention -under MOMI - by infant date of birth.

## Method

### Study setting

The MOMI interventions were conducted in a sub-district of the Kaya health district in the Centre Nord region in Burkina Faso. The Kaya health district comprised 581,521 inhabitants, 52 primary HFs and one regional hospital which serves as a district hospital in 2015 [2]. The study included 12 HFs of the Kaya health district and 69 surrounding communities where the MOMI project was implemented [8]. The implementation of the afore-mentioned activities included workshops/training and supervision of activities in collaboration with the district management team [14].

We calculate the costs incurred by households and health services for maternal PPC before the intervention –January to June 2013- and after the intervention –July 2013 to December 2014-.

For the particular analysis of costs related to the interventions, we looked both at health services and households levels.

### Health services costs

We estimated the annual and the unit cost of health services for the provision of PPC using a bottom up approach. Costs were estimated through a combination of direct observation, using a questionnaire and applying standardized costs from secondary data available in national repositories [15] when direct data were not available.

#### Annual costs

We estimated the annual costs per staff category, building and equipment, and recurrent costs. The significance of the analysis of the cost change, including HFs cost (building, equipment, and salaries) is that the interventions have an effect on the time spent by facility health workers time, also linked to the salaries for PPC delivery, and to the utilisation of rooms and equipment in HFs. The costs are derived from data from the Ministry of health for Kaya health district (unpublished). This source also provided information on buildings, equipment, and salaries at national level.

##### Staff costs: salaries

Staff costs are calculated at district level based on the average monthly salary for every health worker depending on profile for instance for nurses, midwives, auxiliary midwives and auxiliary nurses.

For salaries, we used the mean salary for each health worker profile in Kaya Health district in 2013 - salary levels were not available for other years. While wages of public health workers are revised upwards every two years according to public servant evaluation [16], the increase is usually not immediately effective, we consequently assumed that 2014 salary levels were the same as in 2013.

Since the minimum standards in terms of staffing of a primary HF, usually entail three health workers (one nurse, one auxiliary midwife, and one auxiliary nurse), we calculated the average wage cost of a standard primary HF in Burkina Faso, considering the polyvalence of health workers (see sensitivity analysis (SA)).

##### Building and equipment costs

Norms and standards for the spatial distribution of primary HF buildings and requirements in terms of equipment are elaborated at the national level [17, 18]. As shown in Additional file 1: Figure S1, a typical primary HF would be composed of a maternity ward, a dispensary, a hangar, and accommodations for staff in rural HFs.

The rooms in the maternity ward are commonly used for many interventions related to reproductive health for instance provision of FP, ANC, and PPC. Based on the occupation of rooms (visit room, waiting room) and buildings, and on the normative planning of HF’s architecture [18], we assumed that the occupation rate for the provision of PPC was representing 5% from Jan-June 2013 before the intervention and 10% after the intervention from July–December 2013, in 2014 and in 2015 with the increase of PPC utilization rate.^1^ We assume that the interventions to uptake PPC will contribute to increasing room occupation, having in mind that PPC represent a small share of maternal and reproductive health activities.

While equipment costs were only available for the whole primary HF, we disaggregated them by applying a share of 40% for the dispensary and 60% for the maternity. The basis for the share is the average cost per equipment and the list of equipment per each unit [18].

We applied the annualized cost of the building including equipment and space used for PPC.

##### Recurrent costs

We used the Kaya health district expenditures for reproductive health services from the ministry of health annual action plan financial database for 2013 and 2014 (Additional file 1: Table S1). These costs distinguish themselves from the costs above mentioned as they concern mostly general activities delivery, EmNOC, EPI, immunization campaigns (poliomyelitis, vitamin A) and malaria prevention and treatment. The training expenditures are included in the recurrent costs according to the focus.

##### Discounting

Discounting requires information on the life-cycle of personnel, buildings and equipment and the choice of an appropriate discount rate. We used standardized years of useful life for buildings (20 years), equipment (7 years), and a discount rate of 3% as recommended by most guidelines [17, 19, 20].

##### Sensitivity analysis (SA)

We performed a SA to investigate the potential variation of the parameter values and assumptions in the variation of the output i.e. the total cost of PPC at the health service level [21, 22]. We used plausible uncertainty ranges for the SA per main cost category [23]: staff, infrastructure and recurrent cost.

For staff costs, instead of applying equal distribution of salaries between the three staff members (nurse, auxiliary midwife and auxiliary nurse), we applied the following shares of total salary costs: 40% for the midwife, 40% for the auxiliary midwife and 20% for the nurse. PPC is mainly delivered by midwives and auxiliary midwives.

For infrastructure, we compared what would be the cost if we took the relative share of the whole primary HF building instead of limiting the calculation to the maternity building. For that we applied the proportion of the PPC room surface in the total primary HF to the buildings costs.

For recurrent cost, instead of considering district expenditures, we applied the share of the Kaya health district population in the total population (3%) to the national spending for reproductive health programmes spending from the National Health Accounts (NHA) in 2013 and 2014 for recurrent costs. The baseline figure amounts to 0.1% of the national spending for reproductive health (Additional file 1: Table S2).

#### Health services costs for the provision of one days 6–10 PP visit

We used the annual costs by categories staff, building, equipment and recurrent costs to compute the unit cost of one days 6–10 PP visit.

For personnel unit costs, we used an observation guide to calculate the time attributable to maternal PP services in a primary HF before the intervention [24, 25]. At the MOMI end evaluation study, observations were performed in the course of primary health care activities especially PP check-ups. For staff costs we computed the salary of the health worker who performed the activity using 8 working hours per 5 working days.

We divided the buildings and equipment, and the recurrent costs by the numbers of users for related services at each of the MOMI interventions sites which are 12 primary HFs. Data on the number of users for days 6–10 PPC and number of live births delivery were extracted from the MOMI monitoring database [10, 26].

### Costs at household levels before and after one year intervention by infants date of birth

In this section, we used two household surveys to investigate the economic costs of maternal PP visits before and under MOMI - after one-year implementation of MOMI interventions-.

#### Household study site

The household cross sectional surveys were carried out before and after the intervention in the Kaya health and demographic surveillance site (HDSS). The Kaya HDSS contains 7 urban and 18 rural zones within a radius of 20 km comprising 7 of the 12 primary HFs included in the MOMI project [27]. The Kaya HDSS 2012–2013 routine household data collection – over a period of 6 months – covered 10,629 households including 16,801 women of childbearing age, and 800 newborns and infants. For the survey before the intervention, we selected from the afore-mentioned database a random sample of 840 mothers in their first-year of PP. Eight interviewers performed the data collection using personal digital assistants from December 2012 to January 2013.

For the survey after one year intervention, we selected out of the 2014–2015 Kaya HDSS household database a random sample of 880 mothers in their first-year of PP. Ten interviewers carried out the data collection using personal digital assistants and/or tablets within the Kaya HDSS routine data collection from august 2014 to February 2015.

#### Household cost measurement

We used the same questionnaire on maternal and infant PPC before and after the intervention, which we complemented with additional information from the Kaya HDSS baseline census and updates rounds –not collected routinely- such as individual characteristics and household socio-economic characteristics [27]. Those allowed assessing the comparability of the two samples of mothers before and after the intervention [28].

The questionnaire included information about direct (medical and non-medical-transport costs and modes of transportation of mothers and children to the HF) and indirect costs in terms of the loss of time and productivity using a human capital approach [29]. The interventions were implemented along the continuum of maternal and child PPC: for mothers, first 48 h, days 6–10, days 11–41 and days 42–90; for infants, days 0–5, days 6–10 and days 11–60.

In previous papers, we found that interventions about PPC at days 6–10 were the most successful. The improvement was lower in the later stage of PP when there was a weak integration of maternal PPC to infant immunization services [11, 28, 30]*.* Therefore, we focused the costing study on PPC at days 6–10.

One sample of mothers in their first year of PP were interviewed before the intervention in 2012–13 (757 out of 840: 90%) and another sample after one-year intervention in 2014–15 (754 out of 880: 86%). The mothers are not the same in the two samples therefore the survey is not longitudinal. All the surveys were preceded by staff training, pre-testing, and pilot surveys.

Mothers were asked about the expenditure and time spent for attending maternal PPC, the mode of transportation used to reach the HF, cost and duration of transport. To assess the loss of productivity due to the time dedicated to PPC visits for mothers and infants, we used self-declaration method and asked in the household questionnaires about the income that the mother would have if she had not gone to PPC. However, this is highly subjective. We performed a sensitivity analysis of the opportunity cost using the occupation and applying the minimum agricultural wage for farmers and the guaranteed minimum wage for occupation as small traders. The results are available on request.

We also accounted for the drug and supplies expenditures of women when using PP health services, depending if those were free of charge or purchased for instance at the HF drugstore with a prescription [11, 28]*.*

#### Analysis of household costs

We analysed the data in terms of household cost using the infant date of birth to determine whether the mother and infant were likely to have benefited from the interventions or not. This results in two periods: 1) before the implementation of the intervention (in 2011–2012 and from January to June 2013), and 2) one-year after the implementation (from July to December 2013 and in 2014). We estimated frequencies and means, along with standard errors to analyse the cost of days 6–10 maternal PPC by infant date of birth before and after.

We performed a descriptive analysis with means as well as a two-sample tests of proportions on the before-after linked data using Stata Statistical Software: Release 15 (College Station, TX: Stata Corp LLC).

Costs were estimated in the local currency Franc CFA (XOF) and converted to United States Dollars (USD) following the exchange rate of 1 USD equals 500 Franc CFA for both periods. There was not any noticeable inflation during the study period; therefore, the costs did not require adjustment.

## Results

### Health services cost of PPC

Table 1 presents the yearly costs of PPC by period. The category of recurrent costs was the smallest in both years. Staff and building costs increased after the interventions implementation. The total costs of PPC under MOMI increased substantially (more than doubled).

**Table 1:** Annual cost of PPC by period/year (USD), HF level

	Before		After the interventions			
	Jan-June 2013)		July–December 2013		2014	
	Amount	%	Amount	%	Amount	%
Staff	238	43%	477	45%	477	37%
Buildings and equipments	245	44%	491	47%	491	38%
Recurrent	76	13%	83	8%	338	26%
Total	559	100%	1050	100%	1306	100%

1 USD = 500 FCFA (XOF)

Table 2 shows unit costs of PPC at days 6–10 in the 12 MOMI HFs. The average cost of one PP visit increased from 5 USD in Jan-June 2013 to 8 USD in July-Dec 2013 and then decreased to 3 USD in 2014. The uptake of services may explain this result. In fact, the utilization rate of PP services (number of users / number of live-birth deliveries) improved from 40% (3094 live-birth deliveries) in January–June 2013 to 58% (1167 live-birth deliveries) in July–December 2013, to 74% (4834 live-birth deliveries) in 2014 (Additional file 1: Table S3 Utilisation of PPC at days 6–10 by period/year). The unit-cost was the highest in 2013 (January–June) in Delga (17 USD with 10 users), Damesma and Sector 1 (6 USD with 29 users).

**Table 2:** Unit cost of mother PPC in 2013 and 2014 (USD), HF level

Primary HFs	Before		After the interventions			
	Jan-June 2013		July–December 2013		2014	
	Number of users	Unit cost	Number of users	Unit cost	Number of users	Unit cost
Lebda	228	1.3	140	2.5	279	3.4
Damesma	29	6.1	34	8,8	236	3,9
Delga	10	16,6	10	29.1	304	3.1
Kalambaogo	188	1.4	108	3.1	363	2.7
Basnere	107	2.1	107	3.1	421	2.4
Namsigui	33	5.4	39	7.8	234	4.0
Napalgue	49	3.9	25	11.9	269	3.5
Tangasco	51	3.7	37	8.2	135	6.6
Sector 1	29	6.1	75	4.2	333	2.9
Sector 4	287	1.1	78	3.8	378	2.6
Sector 6	76	2.7	61	7.2	327	3.0
Sector 7	49	3.9	54	5.3	287	3.3
Rural HFs	812	5.1	428	9.3	2241	3.7
Urban HFs	441	3.4	248	5.2	1325	2.9
All MOMI HFs	1253	4.5	676	7.9	3566	3.4

1 USD = 500 FCFA (XOF)

Table S4 (Additional file 1: Table S4) presents the sensitivity analysis (SA) results which are quite similar for SA1 and SA2. SA3 shows different results with the baseline since NHA expenditures in the Kaya Health district encompass all health services expenditures by funding source compared to district expenditures used in the baseline figure.

### Costs of PPC to households

Most mothers do not incur any direct medical costs. However, visiting the HF requires time and travel. Most women walked to the HF (55% before and 53% after the interventions implementation) for PPC. Some used a bicycle (31% before and 28% in 2015) or a motorcycle (10 and 18%). Most interviewed women paid nothing for transportation for days 6–10 PPC (88% before and 72% after). The percentage of interviewed mother who paid for transport increased between the two surveys as well as the transportation cost for maternal PPC. Since the discrepancy cannot be explained by the variability of the sample in terms of socio-economic characteristics [11], it is most likely due to the increase in the utilisation of motorcycles and bicycles between the two survey periods in the study site. Few mothers (share of 3% before and 10% after) were accompanied for the PPC check-ups. Table 3 summarizes the household costs for maternal PPC in USD before and after one year interventions implementation. Direct costs included direct medical and transport costs. Indirect costs are the opportunity costs linked to time spent by mothers and caregivers to visit health services. Total household costs were higher for days 6–10 PPC in 2015 than in 2013 (0.81 versus 0.39 USD; however the observed difference was not significant *P*-value> 0.05) as a result of the increase in medical and indirect costs. As the content of PPC was increased mothers spent more time in the HF explaining the increase in indirect costs. Transport cost increased as explained earlier and the observed difference was significant (P-value < 0.01). Table 4 presents the household mean cost per area of residence and wealth quintile. Household costs were higher in urban than in rural settings (0.24 versus 0.05 USD), and after compared to before the intervention (1.21 after versus 0.60 USD before in urban areas; 0.59 versus 0.05 in rural areas). The mean comparison test after versus before is significant (*p* < 0.05) for transport cost both for rural and urban areas (t = 2.16 and 2.26); and for total household costs in rural areas.

**Table 3:** Household mean cost of mother PPC by infant period of birth (in USD)

Household cost	Before		After one year intervention		Tests of proportions (±Std. Err.)		Mean comparison test after versus before the intervention	
	Mean	SE	Mean	SE	Before	After	t (degrees of freedom)	p
Medical cost	0.03	0.03	0.07	0.06	1.7 (±16.7)	33.6 (±28.0)	0.27 (174)	0.787
Transport cost	0.15	0.06	0.36	0.04	90.8 (±26.9)	186.3 (±19.7)	2.55 (255)	0.011*
Direct cost (Medical cost + transport cost)	0.18	0.07	0.43	0.07	98.5 (±27.6)	211.8 (±30.5)	20.6 (255)	0.039*
Indirect	0.21	0.08	0.37	0.12	84.5 (±24.6)	179.2 (±49.8)	1.1 (±251)	0.263
Total days 6–10 PP visit Household cost (Direct cost + indirect cost)	0.39	0.12	0.81	0.16	180.0 (±39.8)	383.3 (±61.2)	1.9 (258)	0.057

*P-value< 0.05; **P-value< 0.01; ***P-value< 0.001; 1 USD = 500 FCFA (XOF)

**Table 4:** Household mean cost of mother PPC by residence, wealth quintile and by infant period of birth (in USD)

Household cost		Before		After one year intervention		Tests of proportions (±Std. Err.)		Mean comparison test after versus before the intervention	
		Mean	SE	Mean	SE	Before	After	t (degrees of freedom)	p
Areas of residence									
Rural	Medical cost	0.00	0.00	0.02	0.02	0	10.2 (±10.2)	0.35 (97)	0.725
	Transport cost	0.00	0.00	0.32	0.05	34.8 (±34.8)	169.1(±28.1)	2.16 (126)	0.032*
	Direct cost	0.00	0.00	0.34	0.05	34.8 (±34.8)	177.7 (±30.2)	2.14 (126)	0.034*
	Indirect	0.05	0.04	0.24	0.09	16.7 (±9.4)	109.4 (±40.8)	1.10 (123)	0.271
	Total Day 6–10 PP visit Household cost	0.05	0.04	0.59	0.11	50 (±33.9)	283 (±48.6)	2.26 (127)	0.025*
Urban	Medical cost	0.06	0.06	0.00	0.00	29.4 (±29.4)	0	−14.92 (52)	0.141
	Transport cost	0.2	0.09	0.45	0.09	115 (±37)	2144 (±32.8)	19.81 (95)	0.050*
	Direct cost	0.26	0.10	0.45	0.09	127.5 (±38.1)	214 (±32.9)	17.12 (95)	0.090
	Indirect	0.34	0.13	0.76	0.43	128.4 (±38.5)	299.6 (±142.6)	10.07 (95)	0.316
	Total Day 6–10 PP visit Household cost	0.60	0.20	1.21	0.46	252.7 (±58.5)	499.6 (±148.4)	13.46 (97)	0.181
Wealth quintile									
Poorest (Q1)	Medical cost	0.00	0.00	0.00	0.00	0	0	(27)	
	Transport cost	0.08	0.08	0.34	0.10	21.4 (±21.4)	250 (±80.4)	1.98 (40)	0.054*
	Direct cost	0.08	0.08	0.34	0.10	21.4 (±21.4)	250 (±80.4)	1.98 (40)	0.054*
	Indirect	0.32	0.24	0.10	0.31	116.1 (±71.2)	218.8 (±116)	0.60 (40)	0.554
	Total Day 6–10 PP visit Household cost	0.39	0.32	0.84	0.30	137.5 (±91.9)	452.6 (±132.3)	1.56 (41)	0.126
Very poor (Q2)	Medical cost	0.14	0.14	0.00	0.00	71.4 (±71.4)	0	−2 (34)	0.039*
	Transport cost	0.00	0.00	0.30	0.08	33.3 (±33.3)	136.5 (±33.8)	1.75 (53)	0.086
	Direct cost	0.14	0.14	0.30	0.08	66.7 (±45.4)	136.5 (±33.8)	1.13 (53)	0.263
	Indirect	0.00	0.00	0.2931	0.14	15.6 (±15.6)	210.5 (±78.6)	1.59 (52)	0.116
	Total Day 6–10 PP visit Household cost	0.14	0.14	0.60	0.18	78.1 (±44)	336.5 (±91.2)	1.75 (54)	0.085
Poor (Q3)	Medical cost	0.00	0.00	0.05	0.05	0	25 (±25)	0.23 (36)	0.817
	Transport cost	0.00	0.00	0.389	0.08	185.7 (±124.3)	166.7 (±33)	−0.2 (50)	0.844
	Direct cost	0.00	0.00	0.44	0.10	185.7 (±124.3)	186.7 (±42.4)	0.008 (50)	0.993
	Indirect	0.20	0.00	0.06	0.03	28.6 (±18.4)	36.4 (±17.5)	0.17 (49)	0.862
	Total Day 6–10 PP visit Household cost	0.2	0	0.49	0.10	214.3 (±118.4)	222.2 (±43.7)	0.06 (50)	0.947
Richer (Q4)	Medical cost	0.00	0.00	0.00	0.00	0	0	(34)	
	Transport cost	0.13	0.13	0.50	0.10	107.7 (±80.4)	221.6 (±43.2)	1.31 (48)	0.196
	Direct cost	0.13	0.13	0.50	0.10	107.7 (±80.4)	221.6 (±43.2)	1.31 (48)	0.196
	Indirect	0.08	0.08	0.36	0.17	38.5 (±26)	147.2 (±69.2)	0.93 (47)	0.357
	Total Day 6–10 PP visit Household cost	0.22	0.14	0.86	0.22	146.2 (±98.3)	364.9 (±90.6)	1.33 (48)	0.89
Richest (Q5)	Medical cost	0.43	0.20	0.28	0.08	0	133.3 (±133.3)	0.48 (35)	0.635
	Transport cost	0.43	0.20	0.28	0.08	150 (±57.7)	181 (±39.6)	0.42 (56)	0.675
	Direct cost	0.43	0.20	0.55	0.31	150 (±57.7)	276.2 (±110.1)	0.69 (56)	0.492
	Indirect	0.42	0.16	0.78	0.53	181.6 (±68.6)	307.5 (±193.3)	0.42 (55)	0.677
	Total Day 6–10 PP visit Household cost	0.85	0.32	1.33	0.67	322.8 (±93)	569.1 (±233.5)	0.66 (57)	0.512

*P-value< 0,05; **P-value< 0,01; ***P-value< 0,001

Before the intervention, the poorest spent more for PPC than the other quintiles except the richest. After the intervention, the scenario is similar except that the PPC cost for richer and the richest was higher. The mean comparison test after versus before for the poorest quintile is significant (p < 0.05) for transport (t = 1.98 (40) and total cost (t = − 2 (34).

Table 5 shows the mean household costs for integrated services at maternal PPC and at infant immunization at days 6–10 for mothers separating between two interventions: 1) whether mothers were asked about their infant health status and/or whether the infant was examined during maternal PPC at days 6–10 on one hand; 2) whether mothers were asked about their health status and/or were examined at the same occasion as their infant was immunized. Except before the interventions implementation at infant PP visit, household expenditures are higher for the pair mother-Newborn PPC at mother PP visit and for mothers who had their PP check-ups at the same time as their infant was immunized before and after. And, the unit cost of maternal PPC at infant immunization was about 70% higher for mothers whose physical examination was integrated after one year interventions implementation. The increase in households’ costs even for integrated services at infant PP visits may be due to the low integration of services. The mean comparison test after versus before the intervention shows a difference. But the observed difference was significant (*p*-value< 0.05) for those mothers whose infants were not examined at mother PP visit and those who were examined at the infant PP visit.

**Table 5:** Household cost for integrated services at maternal PPC and at infant immunization before and after one-year intervention (in USD)

Integration		Before		After one year interventions		Tests of proportions (±Std. Err.)		Mean comparison test after versus before the intervention	
		Mean	SE	Mean	SE	Before	After	t (degrees of freedom)	p
At mother PP visit									
History taking on infant	Yes	0.42	0.3	0.59	0.27	208.3 (±150.2)	289.2 (±131.1)	0.2 (52)	0.882
	No	0.53	0.36	0.65	0.18	264.3 (±181.5)	327.3 (±87.9)	0.3 (27)	0.737
Infant physical exam	Yes	0.52	0.24	0.76	0.27	258.7 (±118.3)	368.1 (±131.3)	0.3 (91)	0.741
	No	0.3	0.13	0.87	0.12	148.5 (±64)	434.9 (±59)	2.4 (81)	0.018*
At infant PP visit									
History taking on mother	Yes	0.37	0.16	0.84	0.42	158.6 (±61.1)	398.5 (±177.4)	0.8 (68)	0.417
	No	0.41	0.19	0.79	0.14	188.54 (±50.4)	377.9 (±54)	1.9 (188)	0.053
Mother physical exam	Yes	0.25	0.19	1.06	0.6	104.2 (±81.8)	493.3 (±279.4)	0.6 (34)	0.545
	No	0.42	0.15	0.75	0.13	187.5 (±43.01)	363.1 (±51.6)	2 (222)	0.048*

*P-value< 0.05; **P-value< 0.01; ***P-value< 0.001; 1 USD = 500 FCFA (XOF);

## Discussion

The present study assesses the economic costs of PP services for mothers in Kaya health district (Burkina Faso) both at health services and household levels. As days 6–10 PPC were most successful, we chose to perform the costing analysis targeting this visit. Our study found the costs for health services to be lower after the intervention under MOMI than before. This result at supply side is in line with other studies that found that interventions such as integration of services improve impact and reduce costs [13, 31–34]. Indeed, the MOMI project interventions contributed to improve maternal PPC in Kaya health district from 2013 to 2015 [10, 11, 30]*.* However, household mean costs were higher after the intervention. Again, this result is in line with other studies which found that health interventions do make care delivery more effective but also more costly [12]. It is problematic in the context of the population in the Kaya health district who is relatively poor with 47% living under the poverty threshold of 311 USD per year (the national rate was 40% in 2014 [35]). This should be also taken into consideration when evaluating the interventions in terms of the advantages for poor households. Household expenditures for integrated care of mother to child immunization services were also higher when PPC and BCG immunization were integrated although in the context of a weak integration. Indeed, the strength and the intensity of the integration intervention are keys for the effects [12, 26, 36]. This result is probably due to the longer waiting times that contribute to increasing the costs [14]. The financial burden for households would have to be addressed before scaling up this intervention (financing, and sustainability).

Our paper found that most of the interviewed mothers paid nothing for transportation for the PP visit or for infant immunization. This means that transport costs are often not an issue for mothers [37]. Nevertheless, since it is on average the single most important cost item, a minority of women in rural and urban areas and the poorest women paid for transport. Ones should link this finding to equity issues related to transport. Other studies found that equity in access to PP services need to be improved [38, 39]. Further the situation can be extend to all maternal health services since the poorest do not benefit from skilled birth attendants and antenatal care visits [40].

The abolition or reduction of most user fees [41, 42] which contributed to reduce the financial burden of households, benefitted particularly the lower income groups [43–46]. Moreover, following a campaign promise of President Kaboré, free care for mothers and children under 5 years of age was decreed in March 2016 which includes the provision of PPC until week 6 [2]. The financial policy of PPC was a missing piece in the previous subsidy for child birth which covered only delivery and the neonatal period [47]. Undeniably, the policy benefits women and children despite some issues for instance of delays for funding to reach primary HFs and issues of stock-out of drug and supplies during the first year of implementation [48, 49].

The high turnover of staff made it difficult to collect valid information on the number of providers by background and by primary HF in 2013 and 2015 [10, 11, 30]. For this reason, the cost analysis had to rely on the assumption of three standard health workers per primary HF. Moreover, the observation report did not provide enough data for both before and after the MOMI interventions to measure adequately the average activity by staff profile [10]. Check-ups at infant clinics and immunization services were often delivered non-stop by staff with various backgrounds [14]. If all women would come for PPC, the activities of FHW should increase and they should probably face time constraints. However, we acknowledge that we find similar results as Ly’s study on free health care which did not lead to an increase in the workload of FHW as argued by health care providers [50]. Further studies using both direct observation and auto administered time allocation could help in improving cost estimates.

Our study has some limitations. First, as the interventions were designed not with a project concept but with a focus on no additional cost for the health system in the program, we did not address those costs in this paper [8]. We focussed on households and health services perspectives, although the marginal and /or incremental costs of PP services including program costs are of primary concern to policy makers [36].

Second, unfortunately, the case study design does not allow for an alternative cost analysis such as a cost effectiveness study. However, it would be interesting to perform an evaluation of the cost if the target - whether full participation of PP mothers in integrated PPC- had been achieved and whether HFs would have managed to accommodate the increase. We leave this for further research, which should also tackle the long-term effects of the intervention beyond the project implementation time.

Third, we used secondary and retrospective data with possible biases. Fourth, the household survey is a case study and did not control for any factor which may have changed over time. It also did not include a control group that could have helped in assessing the impact of the different interventions on costs but also on the health of infants and mothers, Furthermore, the study is not representative neither of national level and nor of costs in the Kaya health district. However it gives an overview of the trends and components of costs.

## Conclusion

Reproductive health expenditures from many funding sources in Burkina Faso have been growing steadily since 2011 due to the implementation of the Millennium Development Goals and an increase in the subsidies for FP. In this context, the health services costs of maternal PPC at days 6–10 barely decreased after the interventions while the costs to households increased. The financial advantages of some interventions such as the integration of maternal PPC to infant immunization services would require further research for both health services and household levels in the framework of the sustainable development goals (SDGs).

## Supplementary information

**Additional file 1: Table S1.** Expenditure of Kaya health district in 2013 and to 2014 (in USD). **Table S2.** Reproductive health programmes expenditure in Burkina Faso, 2011–2014 (in thousand USD). **Table S3.** Utilisation of PPC at days 6–10 by period/year. **Table S4.** Sensitivity analysis (in USD). **Figure S1.** Example of spatial occupation of a rural primary HF (adapted from [1])

## Data Availability

The datasets used and/or analysed during the current study are available from the corresponding author on reasonable request.

## Abbreviations

ANC: Antenatal care
BCG: Bacillus Calmette-Guérin
EPI: Expanded Program on Immunization
FP: Family planning
GDP: Gross domestic product
HF: Health facility
HFs: Health facilities
Kaya HDSS: Kaya health and demographic surveillance system
MCH: Maternal and child health services
MOMI: Missed opportunities for maternal and infant health
NHA: National Health Accounts
Penta + Hep1: Diphtheria, tetanus, pertussis and poliomyelitis vaccine, haemophilus influenza type B, rotavirus and pneumococcal conjugate vaccine/dose 1
Polio zero: oral poliomyelitis vaccine first dose
PP: Postpartum
PPC: Postpartum care
SA: Sensitivity analysis
USD: United States Dollar

## Footnotes

1 We have considered the real time occupancy of the room corresponding to 100% of time allocated to services for health prevention and promotion delivered during the morning.
