# Supplementary material for: Assessing changes in costs of maternal postpartum services between 2013 and 2014 in Burkina Faso
Source: Int J Equity Health. 2019 Oct 15;18:154. doi: 10.1186/s12939-019-1064-5 (PMC6794858; doi:10.1186/s12939-019-1064-5)
Supplement: Supplementary file 1 — Additional file 1: Table S1. Expenditure of Kaya health district in 2013 and to 2014 (in USD). Table S2. Reproductive health programmes expenditure in Burkina Faso, 2011–2014 (in thousand USD). Table S3. Utilisation of PPC at days 6–10 by period/year. Table S4. Sensitivity analysis (in USD). Figure S1. Example of spatial occupation of a rural primary HF (adapted from [1]) [file 12939_2019_1064_MOESM1_ESM.docx]

**Additional file 1**

**Table S1. Expenditure of Kaya health district in 2013 and to 2014 (in USD)**

| **Expenditure of Kaya health district** | **2013** | | **2014** | |
| --- | --- | --- | --- | --- |
|  | **Amount (USD)** | **%** | **Amount (USD)** | **%** |
| General activities | 1,357,485.366 | 90% | 1,032,746.11 | 85% |
| Reproductive Health | 65,634.752 | 4% | 95,519.198 | 8% |
| Antenatal care |  |  |  |  |
| Delivery and emergency obstetrics care EmONC | 65,634.752 | 4% | 81,655.838 | 7% |
| Family planning |  |  | 5,880.36 | 0% |
| Recurrent activities reproductive health |  |  | 7,983 | 1% |
| Expanded immunization program | 21,834.64 | 1% | 1,3942.7 | 1% |
| Child Malnutrition | 10,585.4 | 1% | 18,011.5 | 1% |
| HIV/prevention of mother-to-child transmission (PMTC) of HIV | 5,349,6 | 0% | 1,329.4 | 0% |
| Performance Based financing |  |  |  |  |
| Immunization campaigns (Poliomyelitis, vitamin A) | 28,769.56 | 2% | 23,281.04 | 2% |
| Malaria prevention and treatment | 26,611.6 | 2% | 12,688 | 1% |
| Other |  |  | 4,956.2 | 0% |
| **Total Kaya** | **1,516,271** | **100%** | **1,218,286** | **100%** |

1 USD= 500 FCFA (XOF)

Table S1 (Supplementary material 1) presents the Kaya health district expenditures by year in 2013 and 2014. Kaya health district expenditures in 2014 were around 1.22 million USD, 20 % lower than in 2013.. General activities have consumed most resources (90%) in 2013. However their share declined to 85% in 2014 due to an increase in reproductive health budget. The share of reproductive health expenditures of reproductive health were composed mainly of expenses linked to the EmONC subsidies (100%) in 2013 and (85.5%) in 2014.

**Table S2. Reproductive health programmes expenditure in Burkina Faso, 2011-2014 (in thousand USD)**

|  | **2011** | | **2012** | | **2013** | | **2014** | |
| --- | --- | --- | --- | --- | --- | --- | --- | --- |
|  | Amount | % | Amount | % | Amount | % | Amount | % |
| **Total health expenditures** | 518,900 | 100% | 559,117 | 100% | 697,418 | 100% | 677,689 | 100% |
| **Reproductive health expenditures** | 27,935 | 5% | 43,258 | 8% | 66,392 | 10% | 72,811 | 11% |
|  |  | * |  | * |  | * |  | * |
| Maternal disease | 23,753 | 85% | 32,019 | 74% | 41,372 | 62% | 48,863 | 67% |
| Perinatal disease | 1,151 | 4% | 3,511 | 8% | 2,393 | 4% | 8,486 | 12% |
| Family planning | 2,198 | 8% | 6,452 | 15% | 9,521 | 14% | 13,827 | 19% |
| Unspecified | 833 | 3% | 1,277 | 3% | 13,107 | 19.74% | 1,634 | 0% |

1 USD=500 FCFA (XOF) * Share in reproductive health

Table S3 presents the utilization rate of PPC by year from January-September 2013 to December 2015 by HFity. Days 6-10 PPC increases from 40% (1250/3094) from Jan-September 2013 to 78% (3748/4804) in year 2015. The increase was substantial in rural (from 50% (812/1613) to 94% (2309/2453) facilities than urban (30% (441/1463) to 61% (1439/2351).

**Table S3. Utilisation of PPC at days 6-10 by period/year**

| **[Primary Health facilities](#_Hlk523328244" \s "1,2593,3502,0,,Primary Health facilities2013 ()** | **[2013 (Jan-September 2013)](#_Hlk523328244" \s "1,2593,3502,0,,Primary Health facilities2013 ()** | | | **[2013 (October-December 2013)](#_Hlk523328244" \s "1,2593,3502,0,,Primary Health facilities2013 ()** | | | **[2014](#_Hlk523328244" \s "1,2593,3502,0,,Primary Health facilities2013 ()** | | |
| --- | --- | --- | --- | --- | --- | --- | --- | --- | --- |
|  | **[Number of user](#_Hlk523328244" \s "1,2593,3502,0,,Primary Health facilities2013 ()** | **[Number of live birth delivery](#_Hlk523328244" \s "1,2593,3502,0,,Primary Health facilities2013 ()** | **[%](#_Hlk523328244" \s "1,2593,3502,0,,Primary Health facilities2013 ()** | **[Number of user](#_Hlk523328244" \s "1,2593,3502,0,,Primary Health facilities2013 ()** | **[Number of live birth delivery](#_Hlk523328244" \s "1,2593,3502,0,,Primary Health facilities2013 ()** | **[%](#_Hlk523328244" \s "1,2593,3502,0,,Primary Health facilities2013 ()** | **[Number of user](#_Hlk523328244" \s "1,2593,3502,0,,Primary Health facilities2013 ()** | **[Number of live birth delivery](#_Hlk523328244" \s "1,2593,3502,0,,Primary Health facilities2013 ()** | **[%](#_Hlk523328244" \s "1,2593,3502,0,,Primary Health facilities2013 ()** |
| **[Lebda](#_Hlk523328244" \s "1,2593,3502,0,,Primary Health facilities2013 ()** | [228](#_Hlk523328244" \s "1,2593,3502,0,,Primary Health facilities2013 () | [219](#_Hlk523328244" \s "1,2593,3502,0,,Primary Health facilities2013 () | [104%](#_Hlk523328244" \s "1,2593,3502,0,,Primary Health facilities2013 () | [33](#_Hlk523328244" \s "1,2593,3502,0,,Primary Health facilities2013 () | [94](#_Hlk523328244" \s "1,2593,3502,0,,Primary Health facilities2013 () | [35%](#_Hlk523328244" \s "1,2593,3502,0,,Primary Health facilities2013 () | [279](#_Hlk523328244" \s "1,2593,3502,0,,Primary Health facilities2013 () | [306](#_Hlk523328244" \s "1,2593,3502,0,,Primary Health facilities2013 () | [91%](#_Hlk523328244" \s "1,2593,3502,0,,Primary Health facilities2013 () |
| **[Damesma](#_Hlk523328244" \s "1,2593,3502,0,,Primary Health facilities2013 ()** | [35](#_Hlk523328244" \s "1,2593,3502,0,,Primary Health facilities2013 () | [150](#_Hlk523328244" \s "1,2593,3502,0,,Primary Health facilities2013 () | [23%](#_Hlk523328244" \s "1,2593,3502,0,,Primary Health facilities2013 () | [28](#_Hlk523328244" \s "1,2593,3502,0,,Primary Health facilities2013 () | [52](#_Hlk523328244" \s "1,2593,3502,0,,Primary Health facilities2013 () | [54%](#_Hlk523328244" \s "1,2593,3502,0,,Primary Health facilities2013 () | [236](#_Hlk523328244" \s "1,2593,3502,0,,Primary Health facilities2013 () | [233](#_Hlk523328244" \s "1,2593,3502,0,,Primary Health facilities2013 () | [101%](#_Hlk523328244" \s "1,2593,3502,0,,Primary Health facilities2013 () |
| **[Delga](#_Hlk523328244" \s "1,2593,3502,0,,Primary Health facilities2013 ()** | [51](#_Hlk523328244" \s "1,2593,3502,0,,Primary Health facilities2013 () | [186](#_Hlk523328244" \s "1,2593,3502,0,,Primary Health facilities2013 () | [27%](#_Hlk523328244" \s "1,2593,3502,0,,Primary Health facilities2013 () | [51](#_Hlk523328244" \s "1,2593,3502,0,,Primary Health facilities2013 () | [77](#_Hlk523328244" \s "1,2593,3502,0,,Primary Health facilities2013 () | [66%](#_Hlk523328244" \s "1,2593,3502,0,,Primary Health facilities2013 () | [304](#_Hlk523328244" \s "1,2593,3502,0,,Primary Health facilities2013 () | [327](#_Hlk523328244" \s "1,2593,3502,0,,Primary Health facilities2013 () | [93%](#_Hlk523328244" \s "1,2593,3502,0,,Primary Health facilities2013 () |
| **[Kalambaogo](#_Hlk523328244" \s "1,2593,3502,0,,Primary Health facilities2013 ()** | [188](#_Hlk523328244" \s "1,2593,3502,0,,Primary Health facilities2013 () | [255](#_Hlk523328244" \s "1,2593,3502,0,,Primary Health facilities2013 () | [74%](#_Hlk523328244" \s "1,2593,3502,0,,Primary Health facilities2013 () | [108](#_Hlk523328244" \s "1,2593,3502,0,,Primary Health facilities2013 () | [107](#_Hlk523328244" \s "1,2593,3502,0,,Primary Health facilities2013 () | [101%](#_Hlk523328244" \s "1,2593,3502,0,,Primary Health facilities2013 () | [363](#_Hlk523328244" \s "1,2593,3502,0,,Primary Health facilities2013 () | [403](#_Hlk523328244" \s "1,2593,3502,0,,Primary Health facilities2013 () | [90%](#_Hlk523328244" \s "1,2593,3502,0,,Primary Health facilities2013 () |
| **[Basnere](#_Hlk523328244" \s "1,2593,3502,0,,Primary Health facilities2013 ()** | [177](#_Hlk523328244" \s "1,2593,3502,0,,Primary Health facilities2013 () | [235](#_Hlk523328244" \s "1,2593,3502,0,,Primary Health facilities2013 () | [75%](#_Hlk523328244" \s "1,2593,3502,0,,Primary Health facilities2013 () | [57](#_Hlk523328244" \s "1,2593,3502,0,,Primary Health facilities2013 () | [93](#_Hlk523328244" \s "1,2593,3502,0,,Primary Health facilities2013 () | [61%](#_Hlk523328244" \s "1,2593,3502,0,,Primary Health facilities2013 () | [421](#_Hlk523328244" \s "1,2593,3502,0,,Primary Health facilities2013 () | [414](#_Hlk523328244" \s "1,2593,3502,0,,Primary Health facilities2013 () | [102%](#_Hlk523328244" \s "1,2593,3502,0,,Primary Health facilities2013 () |
| **[Namsigui](#_Hlk523328244" \s "1,2593,3502,0,,Primary Health facilities2013 ()** | [33](#_Hlk523328244" \s "1,2593,3502,0,,Primary Health facilities2013 () | [256](#_Hlk523328244" \s "1,2593,3502,0,,Primary Health facilities2013 () | [13%](#_Hlk523328244" \s "1,2593,3502,0,,Primary Health facilities2013 () | [39](#_Hlk523328244" \s "1,2593,3502,0,,Primary Health facilities2013 () | [89](#_Hlk523328244" \s "1,2593,3502,0,,Primary Health facilities2013 () | [44%](#_Hlk523328244" \s "1,2593,3502,0,,Primary Health facilities2013 () | [234](#_Hlk523328244" \s "1,2593,3502,0,,Primary Health facilities2013 () | [383](#_Hlk523328244" \s "1,2593,3502,0,,Primary Health facilities2013 () | [61%](#_Hlk523328244" \s "1,2593,3502,0,,Primary Health facilities2013 () |
| **[Napalgue](#_Hlk523328244" \s "1,2593,3502,0,,Primary Health facilities2013 ()** | [49](#_Hlk523328244" \s "1,2593,3502,0,,Primary Health facilities2013 () | [192](#_Hlk523328244" \s "1,2593,3502,0,,Primary Health facilities2013 () | [26%](#_Hlk523328244" \s "1,2593,3502,0,,Primary Health facilities2013 () | [25](#_Hlk523328244" \s "1,2593,3502,0,,Primary Health facilities2013 () | [71](#_Hlk523328244" \s "1,2593,3502,0,,Primary Health facilities2013 () | [35%](#_Hlk523328244" \s "1,2593,3502,0,,Primary Health facilities2013 () | [269](#_Hlk523328244" \s "1,2593,3502,0,,Primary Health facilities2013 () | [300](#_Hlk523328244" \s "1,2593,3502,0,,Primary Health facilities2013 () | [90%](#_Hlk523328244" \s "1,2593,3502,0,,Primary Health facilities2013 () |
| **[Tangasco](#_Hlk523328244" \s "1,2593,3502,0,,Primary Health facilities2013 ()** | [51](#_Hlk523328244" \s "1,2593,3502,0,,Primary Health facilities2013 () | [138](#_Hlk523328244" \s "1,2593,3502,0,,Primary Health facilities2013 () | [37%](#_Hlk523328244" \s "1,2593,3502,0,,Primary Health facilities2013 () | [37](#_Hlk523328244" \s "1,2593,3502,0,,Primary Health facilities2013 () | [43](#_Hlk523328244" \s "1,2593,3502,0,,Primary Health facilities2013 () | [86%](#_Hlk523328244" \s "1,2593,3502,0,,Primary Health facilities2013 () | [135](#_Hlk523328244" \s "1,2593,3502,0,,Primary Health facilities2013 () | [130](#_Hlk523328244" \s "1,2593,3502,0,,Primary Health facilities2013 () | [104%](#_Hlk523328244" \s "1,2593,3502,0,,Primary Health facilities2013 () |
| **[Sector 1](#_Hlk523328244" \s "1,2593,3502,0,,Primary Health facilities2013 ()** | [29](#_Hlk523328244" \s "1,2593,3502,0,,Primary Health facilities2013 () | [508](#_Hlk523328244" \s "1,2593,3502,0,,Primary Health facilities2013 () | [6%](#_Hlk523328244" \s "1,2593,3502,0,,Primary Health facilities2013 () | [75](#_Hlk523328244" \s "1,2593,3502,0,,Primary Health facilities2013 () | [210](#_Hlk523328244" \s "1,2593,3502,0,,Primary Health facilities2013 () | [36%](#_Hlk523328244" \s "1,2593,3502,0,,Primary Health facilities2013 () | [333](#_Hlk523328244" \s "1,2593,3502,0,,Primary Health facilities2013 () | [783](#_Hlk523328244" \s "1,2593,3502,0,,Primary Health facilities2013 () | [43%](#_Hlk523328244" \s "1,2593,3502,0,,Primary Health facilities2013 () |
| **[Sector 4](#_Hlk523328244" \s "1,2593,3502,0,,Primary Health facilities2013 ()** | [287](#_Hlk523328244" \s "1,2593,3502,0,,Primary Health facilities2013 () | [287](#_Hlk523328244" \s "1,2593,3502,0,,Primary Health facilities2013 () | [100%](#_Hlk523328244" \s "1,2593,3502,0,,Primary Health facilities2013 () | [35](#_Hlk523328244" \s "1,2593,3502,0,,Primary Health facilities2013 () | [88](#_Hlk523328244" \s "1,2593,3502,0,,Primary Health facilities2013 () | [40%](#_Hlk523328244" \s "1,2593,3502,0,,Primary Health facilities2013 () | [378](#_Hlk523328244" \s "1,2593,3502,0,,Primary Health facilities2013 () | [480](#_Hlk523328244" \s "1,2593,3502,0,,Primary Health facilities2013 () | [79%](#_Hlk523328244" \s "1,2593,3502,0,,Primary Health facilities2013 () |
| **[Sector 6](#_Hlk523328244" \s "1,2593,3502,0,,Primary Health facilities2013 ()** | [76](#_Hlk523328244" \s "1,2593,3502,0,,Primary Health facilities2013 () | [352](#_Hlk523328244" \s "1,2593,3502,0,,Primary Health facilities2013 () | [22%](#_Hlk523328244" \s "1,2593,3502,0,,Primary Health facilities2013 () | [61](#_Hlk523328244" \s "1,2593,3502,0,,Primary Health facilities2013 () | [135](#_Hlk523328244" \s "1,2593,3502,0,,Primary Health facilities2013 () | [45%](#_Hlk523328244" \s "1,2593,3502,0,,Primary Health facilities2013 () | [327](#_Hlk523328244" \s "1,2593,3502,0,,Primary Health facilities2013 () | [628](#_Hlk523328244" \s "1,2593,3502,0,,Primary Health facilities2013 () | [52%](#_Hlk523328244" \s "1,2593,3502,0,,Primary Health facilities2013 () |
| **[Sector 7](#_Hlk523328244" \s "1,2593,3502,0,,Primary Health facilities2013 ()** | [49](#_Hlk523328244" \s "1,2593,3502,0,,Primary Health facilities2013 () | [316](#_Hlk523328244" \s "1,2593,3502,0,,Primary Health facilities2013 () | [16%](#_Hlk523328244" \s "1,2593,3502,0,,Primary Health facilities2013 () | [54](#_Hlk523328244" \s "1,2593,3502,0,,Primary Health facilities2013 () | [108](#_Hlk523328244" \s "1,2593,3502,0,,Primary Health facilities2013 () | [50%](#_Hlk523328244" \s "1,2593,3502,0,,Primary Health facilities2013 () | [287](#_Hlk523328244" \s "1,2593,3502,0,,Primary Health facilities2013 () | [447](#_Hlk523328244" \s "1,2593,3502,0,,Primary Health facilities2013 () | [64%](#_Hlk523328244" \s "1,2593,3502,0,,Primary Health facilities2013 () |
| **[Rural HFs](#_Hlk523328244" \s "1,2593,3502,0,,Primary Health facilities2013 ()** | [812](#_Hlk523328244" \s "1,2593,3502,0,,Primary Health facilities2013 () | [1631](#_Hlk523328244" \s "1,2593,3502,0,,Primary Health facilities2013 () | [50%](#_Hlk523328244" \s "1,2593,3502,0,,Primary Health facilities2013 () | [428](#_Hlk523328244" \s "1,2593,3502,0,,Primary Health facilities2013 () | [626](#_Hlk523328244" \s "1,2593,3502,0,,Primary Health facilities2013 () | [68%](#_Hlk523328244" \s "1,2593,3502,0,,Primary Health facilities2013 () | [2241](#_Hlk523328244" \s "1,2593,3502,0,,Primary Health facilities2013 () | [2496](#_Hlk523328244" \s "1,2593,3502,0,,Primary Health facilities2013 () | [90%](#_Hlk523328244" \s "1,2593,3502,0,,Primary Health facilities2013 () |
| **[Urban HFs](#_Hlk523328244" \s "1,2593,3502,0,,Primary Health facilities2013 ()** | [441](#_Hlk523328244" \s "1,2593,3502,0,,Primary Health facilities2013 () | [1463](#_Hlk523328244" \s "1,2593,3502,0,,Primary Health facilities2013 () | [30%](#_Hlk523328244" \s "1,2593,3502,0,,Primary Health facilities2013 () | [248](#_Hlk523328244" \s "1,2593,3502,0,,Primary Health facilities2013 () | [541](#_Hlk523328244" \s "1,2593,3502,0,,Primary Health facilities2013 () | [46%](#_Hlk523328244" \s "1,2593,3502,0,,Primary Health facilities2013 () | [1325](#_Hlk523328244" \s "1,2593,3502,0,,Primary Health facilities2013 () | [2338](#_Hlk523328244" \s "1,2593,3502,0,,Primary Health facilities2013 () | [57%](#_Hlk523328244" \s "1,2593,3502,0,,Primary Health facilities2013 () |
| **[All MOMI HFs](#_Hlk523328244" \s "1,2593,3502,0,,Primary Health facilities2013 ()** | [1253](#_Hlk523328244" \s "1,2593,3502,0,,Primary Health facilities2013 () | [3094](#_Hlk523328244" \s "1,2593,3502,0,,Primary Health facilities2013 () | [40%](#_Hlk523328244" \s "1,2593,3502,0,,Primary Health facilities2013 () | [676](#_Hlk523328244" \s "1,2593,3502,0,,Primary Health facilities2013 () | [1167](#_Hlk523328244" \s "1,2593,3502,0,,Primary Health facilities2013 () | [58%](#_Hlk523328244" \s "1,2593,3502,0,,Primary Health facilities2013 () | [3566](#_Hlk523328244" \s "1,2593,3502,0,,Primary Health facilities2013 () | [4834](#_Hlk523328244" \s "1,2593,3502,0,,Primary Health facilities2013 () | [74%](#_Hlk523328244" \s "1,2593,3502,0,,Primary Health facilities2013 () |

**Table S4. Sensitivity analysis (in USD)**

| **Sensitivity analysis (SA)** | **Total cost** | | | **% change** | | |
| --- | --- | --- | --- | --- | --- | --- |
|  | **Jan-June 2013** | **July-December 2013** | **2014** | **Jan-June 2013)** | **July-December 2013** | **2014** |
| **Baseline figure** | **559,36** | **1050,30** | **1306,18** | **0%** | **0%** | **0%** |
| **SA1** | **594,11** | **1119,79** | **1375,67** | **6%** | **6%** | **5%** |
| **SA2** | **478,02** | **887,61** | **1143,50** | **-24%** | **-26%** | **-20%** |
| **SA3** | **90199,23** | **180398,47** | **197747,26** | **99%** | **100%** | **99%** |

Table S4 (Supplementary material 4) presents the sensitivity analysis (SA) results which are quite similar for SA1 and SA2. SA3 shows different results with the baseline since NHA expenditures in the Kaya Health district encompass all health services expenditures by funding source compared to district expenditures used in the baseline figure.


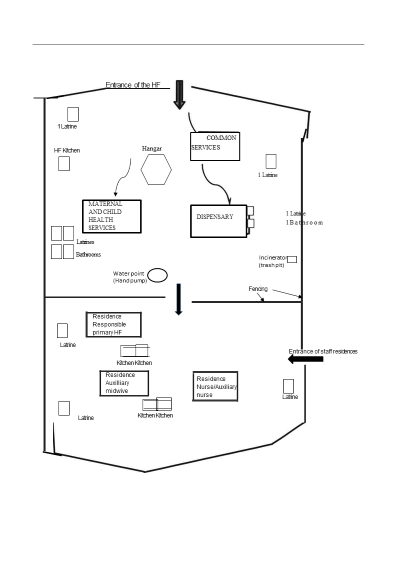


**Figure S1. Example of spatial occupation of a Rural primary HF (adapted from** [1])

1, Direction des Infrastructures des Equipements et de la Maintenance (DIEM), *Normes et standards en infrastructures et en equipements du Centre de Santé et de Promotion Sociale, du Centre Médical avec Antenne Chirurgical et du Centre Hospitalier Régional, Ministe`re de la Santé, Burkina Faso,* 2004,
